# Associations of Thyroid and Parathyroid Hormones with Arterial Stiffness in Emergency Department Patients: A Prospective Cross-Sectional Study

**DOI:** 10.3390/medicina61050812

**Published:** 2025-04-28

**Authors:** Roman Brock, Andrea Kornfehl, Julia Oppenauer, Felix Eibensteiner, Marco Neymayer, Christoph Veigl, Carina Cuhaj, Oliver Erbes, Sophia Wirth, Thomas Perkmann, Helmuth Haslacher, Markus Müller, Oliver Schlager, Peter Wolf, Sebastian Schnaubelt

**Affiliations:** 1 Department of Emergency Medicine, Medical University of Vienna, 1090 Vienna, Austria; 2 Department of Biomedical Imaging and Image-Guided Therapy, Medical University of Vienna, 1090 Vienna, Austria; 3 Department of Laboratory Medicine, Medical University of Vienna, 1090 Vienna, Austria; 4 Division of Angiology, Department of Internal Medicine II, Medical University of Vienna, 1090 Vienna, Austria; 5 Division of Endocrinology, Department of Internal Medicine III, Medical University of Vienna, 1090 Vienna, Austria; 6 Emergency Medical Service Vienna, 1030 Vienna, Austria

**Keywords:** pulse wave velocity, vascular calcification, arterial stiffness, thyroid dysfunction, parathyroid hormone, vitamin D

## Abstract

*Background and Objectives:* Cardiovascular diseases are prevalent entities, especially in emergency patients. Arterial stiffness is a known predictor of cardiovascular risk and mortality and is quantified by carotid-femoral pulse wave velocity (cfPWV). It is caused in part by vascular calcification, but exact details of the underlying mechanisms are yet to be elucidated, and current data suggest endocrine influences. This study thus aimed to assess the associations of endocrine parameters, particularly thyroid and parathyroid hormones, calcium, inorganic phosphate, and vitamin D, with cfPWV as a surrogate for arterial stiffness. *Materials and Methods:* Adults presenting to a single tertiary care emergency department in Vienna between 2018 and 2023 were prospectively enrolled. CfPWV was measured non-invasively, and levels of thyroid and parathyroid hormones and 25-hydroxyvitamin D, calcium, and inorganic phosphate were assessed. *Results:* In total, data from 827 patients, predominantly male (57%) and around 60 (47–72) years of age, were assessed. We observed a significant worsening of cfPWV with increasing parathyroid hormone levels (*p* < 0.001) and TSH levels (*p* = 0.03). No significant influences of calcium, inorganic phosphate, or 25-hydroxyvitamin D were observed. *Conclusions:* Thyroid and parathyroid hormone levels are associated with arterial stiffness in emergency department patients, suggesting a need for a comprehensive workup in patients at risk because of comorbidities and age. Additional prospective studies are needed to further elucidate the role of endocrinology in arterial stiffness and the subsequent relevance in emergency medicine.

## 1. Background

Endotheliopathy and arterial disease are common problems in modern societies, and increased arterial stiffness is a predictor of cardiovascular risk and mortality [1,2,3]. It favors, among others, the development of coronary artery disease and heart failure and increases the risk of stroke [1]. One of the many factors associated with increased arterial stiffness is vascular calcification influenced by vascular smooth muscle cells and their extracellular matrix [3,4,5]. The exact process behind vascular calcification is not yet fully understood, but current knowledge suggests links to osteogenesis and bone metabolism [4]. Involved mechanisms include pathways influenced by parathyroid hormone (iPTH), possibly modulating vascular calcification [6,7] due to an imbalance in calcium and phosphate homeostasis [4]. Vitamin D deficiency as well as hyperparathyroidism have been connected to cardiovascular disease (e.g., hypertension and atherosclerosis) and renal dysfunction [8,9]. Deficiency in Vitamin D, but also exogenous excess, were shown to have a detrimental effect on the vascular system and atherosclerosis [9,10]. Therefore, assessment of these parameters may play a role in an individualized risk assessment approach. Additionally, links between thyroid hormones and vascular stiffness are emerging, and so far, these have been explained via various mechanisms, including direct nuclear and extranuclear interactions, catecholamine sensitivity, and nitric oxide (NO) production [11,12,13,14,15,16]. Furthermore, impaired glucose metabolism is linked to increased arterial stiffness as well [17,18,19,20,21,22,23].

Contrary to the exact pathophysiological mechanisms, quantifying arterial stiffness is relatively simple. Many different methods are available, including pulse wave velocity, where the non-invasive measurement of carotid-femoral pulse wave velocity (cfPWV) is considered a high standard due to its reliability [24]. It can be calculated quickly and cost-effectively utilizing four sphygmomanometers around the patient’s upper arms and the lower limbs above the ankle. Despite the benefits, it has to be mentioned that cfPWV is directly correlated with heart rate and age [25]. We previously identified relevant associations between pulse wave velocity and acute medical conditions such as acute coronary syndrome [26] or COVID-19 [27]. Because of the limited and partly conflicting data on the topic, as well as the relevance and prevalence of associated diseases in acutely ill patients, this study aimed to elucidate the associations of the endocrine and vascular system, specifically the thyroid and parathyroid influence and the impact of glucose metabolism on vascular stiffness in an emergency patient collective.

## 2. Methods

### 2.1. Study Population

Consenting adults (>18 years of age) presenting to the Department of Emergency Medicine of the Medical University of Vienna, Austria, a tertiary care center, between November 2018 and October 2023 were prospectively enrolled in this observational study if inclusion did not result in a delay of life-saving medical interventions (hemodynamical or respiratory unstable patients). Patients were excluded in case of pregnancy or if inclusion was not feasible due to urgent medical interventions or diagnostics. Otherwise, no specific exclusion criteria were applied. This study was approved by the Ethics Committee of the Medical University of Vienna, Austria (2197/2017), and conducted according to good clinical practice guidelines.

### 2.2. Study-Related Procedures

Patient characteristics and clinical and demographic data were recorded at study inclusion. Comorbidities were collected using a standardized questionnaire at the same time. Afterward, patients received oscillometric PWV measurements with a certified BOSO ABI Systems 100 PWV^®^ device (Bosch&Sohn GmbH, Jungingen, Germany) if feasible. CfPWV measurements were performed by the study author group utilizing sphygmomanometer cuffs around the subject’s extremities (upper arms, lower limbs) while in a supine position after a resting period of ten minutes. The patient’s height was considered for calculating cfPWV based on the time shift of captured pulse waves. The precision was assessed upfront by measuring the cfPWV in ten volunteers repeatedly, yielding a mean variation of 2.8%.

Additionally, blood samples were drawn, and laboratory analyses were performed by the Department of Laboratory Medicine, Medical University of Vienna, Austria. Levels of intact parathyroid hormone (iPTH; normal range of 15–65 pg/mL), thyroid-stimulating hormone (TSH; normal range of 0.51–4.3 µIU/mL between 11 and 20 years of age and 0.27–4.2 µIU/mL above 20 years), free T3 (fT3; normal range of 2.28–5.01 pg/mL between 11 and 19 years and 2.15–4.12 pg/mL above 19 years), and free T4 (fT4; normal range of 0.93–1.60 ng/dL between 11 and 19 years and 0.76–1.66 ng/dL above 19 years) were assessed using electrochemiluminescence immunoassays. Levels of 25-hydroxyvitamin D (25(OH)D) were assessed using chemiluminescence immunoassays, with a normal range of 75–250 nmol/L. Serum calcium concentration was assessed with the o-cresolphthalein complexone method. Normal ranges are age-dependent, with 2.15–2.50 mmol/L for 18–59 years, 2.20–2.55 mmol/L for 59–90 years, and 2.05–2.40 mmol/L above 90 years. Inorganic phosphate was assessed with molybdate reaction, with normal ranges of 0.94–1.55 mmol/L for 16–19 years and 0.81–1.45 mmol/L above 19 years of age. Serum glucose levels were measured with the hexokinase method, with a normal range of 60–100 mg/dL from 1 month to 19 years and 74–109 mg/dL above 19 years [28].

### 2.3. Statistical Analysis

The primary endpoints of this study were the association of cfPWV with different levels (terciles) of iPTH and 25(OH)D as well as the influence of thyroid dysfunction. Additionally, serum concentrations of calcium, as well as inorganic phosphate and serum glucose, were assessed. Descriptive outcomes include associations between cfPWV and typical cardiovascular comorbidities in the present collective.

Variables are presented as absolute numbers (*n*), relative frequencies (%), and medians with interquartile ranges (IQRs). Group-wise comparisons of metric variables were performed with the Wilcoxon test for independent variables or the Kruskal–Wallis test. Nominal variables were assessed with the χ^2^-test. Linear regression models were calculated to compensate for confounders (age and heart rate). The statistical analysis was conducted with R 4.3.3 (R Foundation for Statistical Computing, Vienna, Austria), and *p*-values < 0.05 were considered statistically significant.

## 3. Results

In total, 918 patients were included. The most common chief complaints included thoracic pain (*n* = 599, 65.3%), suspicion for COVID-19 (*n* = 104, 11.3%) or pulmonary embolism (*n* = 64, 7%), dyspnea (*n* = 60, 6.5%), renal insufficiency (*n* = 28, 3.1%) and atrial fibrillation (*n* = 20, 5.5%). Viable cfPWV measurements without artifacts were available in 827 patients (90%). Of these, patients were 60 (47–72) years of age on average and predominantly male (*n* = 473, 57%). The median iPTH serum level was 42.2 (30.8–60.4) pg/mL. Cutoffs for tercile assignment were 34.9 pg/mL and 54.6 pg/mL. The median serum level of 25-hydroxyvitamin D was 54.6 (31.7–77.1) nmol/L, and 2.31 (2.23–2.37) mmol/L for calcium. The median level of inorganic phosphate was 0.990 (0.87–1.12) mmol/L. A total of 122 patients exceeded the upper limit of iPTH (65 pg/mL). Of these, four patients (3.3%) showed elevated calcium levels above the corresponding thresholds (suspicion for primary hyperparathyroidism). The median 25-hydroxyvitamin D level in this group was 36.3 (23.2–58.7) nmol/L. Patient characteristics are shown in Table 1.

**Figure 1:**
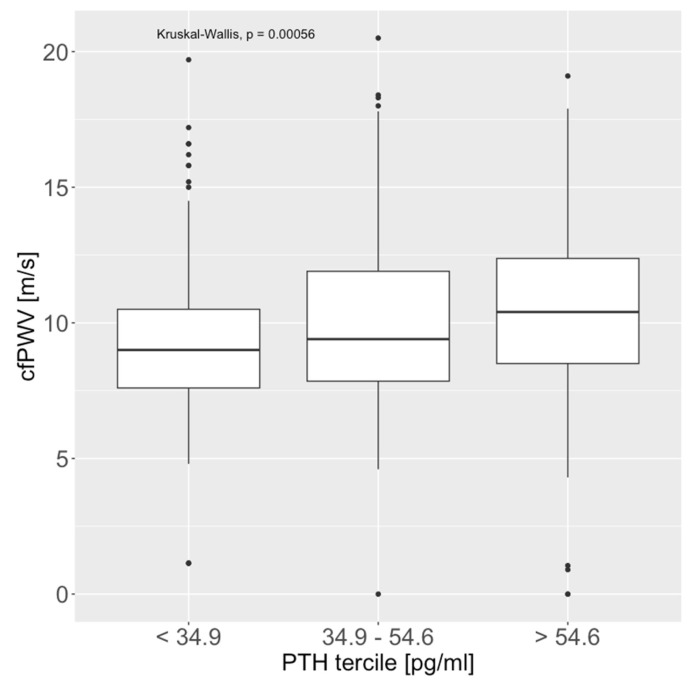
Carotid-femoral pulse wave velocity (cfPWV) stratified by parathyroid hormone (PTH) terciles.

CfPWV was significantly associated with iPTH terciles (first tercile: 9 (7.6–10.5) m/s; second tercile: 9.4 (7.85–11.9) m/s; third tercile: 10.4 (8.5–12.375) m/s; *p* < 0.001, Figure 1). After correcting for age and heart rate (both do not correlate well with iPTH; R_heart_rate_ = 0.017, R_age_ = 0.19), the upper tercile remained significantly associated with an increase in cfPWV (*p* = 0.0177). A trend towards higher cfPWV within higher iPTH tertiles remains visible in a subgroup analysis based on comorbidities, especially in the coronary, peripheral, and cerebrovascular disease subgroup (see Supplementary Figure S2). The 25(OH)D3 levels did not correlate significantly with cfPWV, and there was no significant impact of albumin-corrected calcium or inorganic phosphate levels on cfPWV.

Data on thyroid function, as well as cfPWV, were available in 669 patients. Regarding thyroid dysfunction, our results suggest a positive association of cfPWV with TSH levels (*p* = 0.03) after correcting for age and heart rate (both do not correlate well with TSH; R_heart_rate_ = −0.03, R_age_ = 0.049). However, no significant association between cfPWV and free serum levels of T3 or T4 was observed. An overview of measured thyroid hormone levels and cfPWV is shown in Table 2.

**Figure 2:**
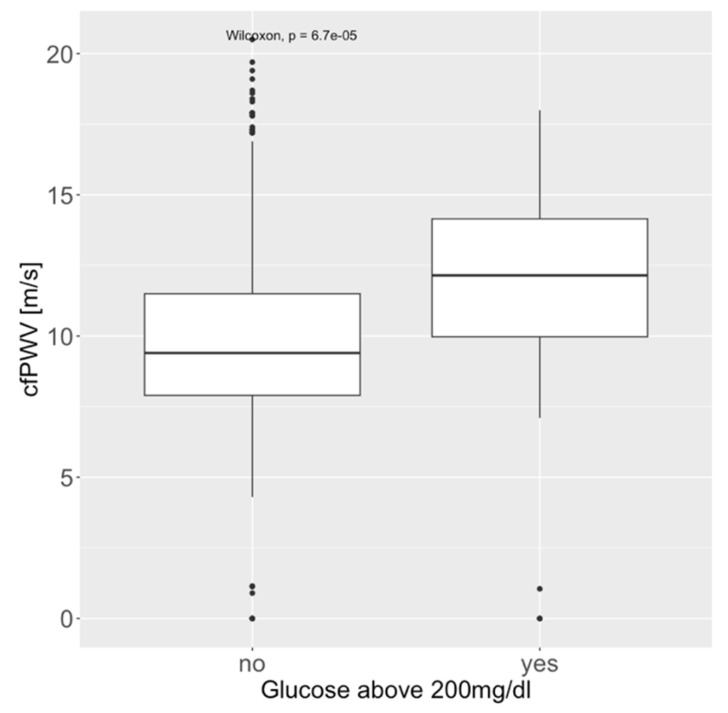
Association of carotid-femoral pulse wave velocity (cfPWV) and serum glucose above 200 mg/dL.

**Figure 3:**
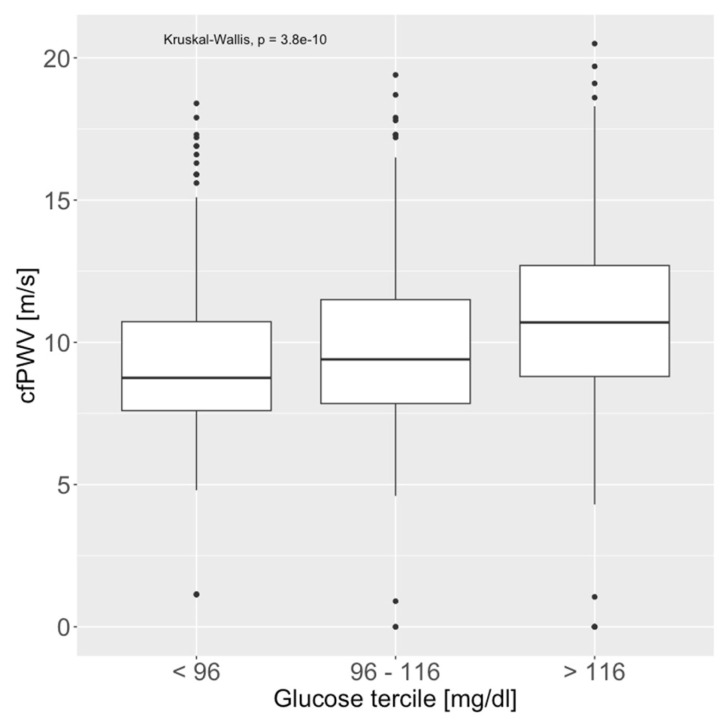
Association of serum glucose levels (terciles) and carotid-femoral pulse wave velocity (cfPWV).

Data on serum glucose levels and cfPWV were available in 763 patients. Median serum glucose was 102 (91–124) mg/dL. Serum glucose measured at a singular point in time was significantly associated with cfPWV (*p* < 0.001, Figure 2 and Figure 3). The highest tercile (above 116 mg/dL, *p* < 0.001), as well as glucose above 200 mg/dL (*p* = 0.004), remained highly significant after correcting for age and heart rate (both do not correlate well with glucose levels; R_heart_rate_ = 0.17, R_age_ = 0.28). Supplementary Figures S3 and S4 show the analysis of these effects in subgroups based on comorbidities.

Typical cardiovascular comorbidities, including coronary and peripheral artery disease, cerebrovascular disease, hypertension, dyslipidemia, diabetes mellitus type 2, and chronic kidney disease, were associated with higher cfPWV values (Supplementary Figure S1). Regarding kidney function, our analysis shows a small but significant correlation of cfPWV with creatinine (R = 0.12, slope = 0.36, *p* = 0.0012). However, this association is not visible with glomerular filtration rate (R = −0.046, slope = −0.011, *p* = 0.55).

## 4. Discussion

Because of the high prevalence and relevance of vascular disease and stiffening, respective mechanisms and influencing circumstances are of interest. The importance of arterial disease in general and associated morbidities [1] is even more pronounced in acutely ill patients, since a better or worse vascular state provides a better or worse resilience and base of the respective patients in emergency conditions. For instance, vascular function has been found to be impaired in infection, to be associated with coronary pathologies, and to be associated with short- and long-term mortality in emergency department patients [26,27,29]. In the present study, we focused on endocrinological laboratory parameters to potentially shine additional light on the underlying mechanisms of vascular dysfunction in acute illness. We tried to assess the influence of thyroid, parathyroid, and glucose levels on cfPWV as surrogates for arterial stiffness.

Our data show a worsening of arterial stiffness with increasing levels of iPTH. The underlying pathophysiological mechanisms are not yet fully understood, but the literature suggests an involvement of the Wnt/ß-catenin pathway and RANK/RANKL interaction, as well as modulation by calcium, phosphorous, or vitamin D [30,31,32,33,34]. Levels of calcium or inorganic phosphate as well as 25(OH) vitamin D at the time of measurement did not reveal any clues in terms of cfPWV dynamics in the present study, probably reflecting the inappropriateness of a singular measurement as a surrogate for the burden over time. However, the relevant time frame for alterations in arterial stiffness is yet to be determined [35].

Much research has been conducted regarding vitamin D, with heterogeneous conclusions. Fortier et al. observed a significant but minimal 25(OH)D influence on cfPWV in an unadjusted model in a small (*n* = 85) hemodialysis collective, but contrary to our data, they did not observe an influence of iPTH [36]. Also, vitamin D administration has been reported to be linked to accelerated vascular calcification in rats [37]. Still, an association of higher 25(OH)D levels with lower calcification of coronary arteries in humans has indeed been shown [38]. More extensive studies suggest a U-shaped association between vitamin D and vascular events or mortality [39,40]. Additionally, Durup et al. were able to show a significant increase in all-cause mortality with increasing iPTH levels by assessing the database of the Copenhagen General Practitioners Laboratory between 2004 and 2010, the only laboratory serving general practitioners in the area of Copenhagen at the time (*n* = 34,996) [39]. It remains unknown if this increase in all-cause mortality is attributable, at least in part, to an increased vascular calcification.

Thyroid hormones influence the vascular system via various mechanisms. They exert more direct effects by modulating sensitivity to catecholamines [15] and NO production [11,13], and they also have a wide range of effects mediated by integrin-associated extracellular receptors [14]. Moreover, thyroid dysfunction may also indirectly impact the vascular system by influencing blood pressure (influencing vascular resistance by nitric oxide and calcium reuptake modulation as well as cardiac output) [41], lipid levels (modulation of LDL receptor density, cholesterol 7 alpha c oxide, calcium reuptake, and cardiac output), lipid levels (modulation of LDL receptor density and cholesterol 7 alpha-hydroxylase) [41,42,43] and insulin resistance [44,45,46,47,48].

Regarding thyroid function, an abundance of data is available, showing increased arterial stiffness in both directions of thyroid dysfunction. Mousa et al. demonstrated a significant association of overt and subclinical hypothyroidism with PWV compared to euthyroid controls and attributed this effect to altered lipid levels [44]. Wang J et al. also showed an inverse association of fT4 levels with brachial-ankle PWV [49]. Wang P et al. were also able to demonstrate an inverse connection between fT3 levels and prevalent peripheral artery disease (defined by ankle-brachial index) [50]. However, Grove-Laugesen et al. found an increase in PWV in hyperthyroid patients with Graves’ disease (morbus Basedow) compared to euthyroid controls [51]. In accordance, Yildiz et al. showed that this association is present in overt and latent hyperthyroid dysfunction [52]. A recent meta-analysis revealed an increase in PWV in overt and subclinical hypothyroid patients and also showed worsened arterial stiffness in patients with thyrotoxicosis [53].

Further, our data show associations of disturbed glucose metabolism with arterial stiffness, consistent with the previous literature [17,18,19,20,21,22,23]. A history of diabetes as well as serum glucose levels at a singular point of time independent of fasting state (terciles as well as exceeding the limit of 200 mg/dL) were significantly associated with higher cfPWV, even after correction for age and heart rate. This finding emphasizes the connection of glucose metabolism to the macro- and microvascular system [54,55,56,57,58,59], especially in emergency patients. Of note, no data on fasting glucose levels, glucose tolerance, or HbA1c could be collected, thereby limiting the value of this association. However, the strength of the observed association, despite the values only being a one-time measurement, underlines the potential of further research into this topic—maybe peak glucose levels could lead to sudden changes in vascular function, impacting acute illness.

In summary, our data show clear associations of iPTH, serum glucose levels, and thyroid hormones with arterial stiffness, highlighting the need for a comprehensive workup, especially in patients at higher risk due to comorbidities and age. In-depth assessment of mentioned endocrine factors could provide a path to a more patient-centered approach of personalized medicine by aiding individual risk assessment and therefore possibly enabling closer follow-up or preventive strategies. These findings should be taken into account for further research into vascular function in acute illness.

### Limitations

The limitations of the present study primarily arise from the missing data, probably leading to selection bias. Additionally, data quality on medication use was unsatisfying due to changes in the questionnaires and lack of documentation quality. The current study population is based on an emergency patient collective; therefore, applicability to other cohorts has to be assessed carefully. Patients included in the study presented with acute medical conditions partially founded on chronic diseases. This could result in various confounding factors. Finally, no follow-ups or repeated measurements were collected.

## 5. Conclusions

Elevated iPTH, serum glucose levels, and thyroid dysfunction influence arterial stiffness in acutely ill patients. Closer endocrinological monitoring may benefit individuals at risk because of their comorbidities and age, and relevant subpopulations in emergency departments being at special cardiovascular risk could potentially be identified. However, prospective studies are needed to further elucidate the role of endocrinology in arterial stiffness and the subsequent relevance and opportunities for action in emergency medicine.

## Figures and Tables

**Table 1:** Baseline characteristics stratified by carotid-femoral pulse wave velocity (cfPWV) terciles. BMI = body-mass index; iPTH = parathyroid hormone; 25(OH)D = 25-hydroxyvitamin D. Bold *p*-values indicate statistically significant values.

	cfPWV < 8.4 m/s (*n* = 280)	cfPWV 8.4–10.9 m/s (*n* = 275)	cfPWV > 10.9 m/s (*n* = 272)	Overall (*n* = 827)	*p*-Value
**Age**					**<0.001**
Mean (SD)	47.6 (16.5)	57.8 (13.6)	71.7 (12.1)	58.9 (17.3)	
Median (Q1, Q3)	46.0 (36.0, 58.3)	58.0 (49.0, 67.0)	73.5 (65.0, 80.0)	60.0 (47.0, 72.0)	
**Sex**					0.727
female	124 (44.3%)	113 (41.1%)	116 (42.6%)	353 (42.7%)	
male	155 (55.4%)	162 (58.9%)	156 (57.4%)	473 (57.2%)	
Missing	1 (0.4%)	0 (0%)	0 (0%)	1 (0.1%)	
**BMI**					**0.004**
Mean (SD)	27.1 (5.74)	28.2 (5.33)	26.7 (4.75)	27.3 (5.32)	
Median (Q1, Q3)	26.3 (22.9, 30.4)	27.3 (24.5, 31.2)	26.0 (23.2, 29.4)	26.6 (23.5, 30.4)	
Missing	0 (0%)	0 (0%)	2 (0.7%)	2 (0.2%)	
**iPTH**					**<0.001**
Mean (SD)	45.4 (27.9)	62.9 (148)	63.8 (56.7)	57.1 (93.0)	
Median (Q1, Q3)	39.8 (29.5, 51.6)	40.9 (29.9, 60.7)	47.4 (35.2, 69.8)	42.2 (30.8, 60.4)	
Missing	107 (38.2%)	109 (39.6%)	111 (40.8%)	327 (39.5%)	
**25(OH)D3**					0.3
Mean (SD)	55.5 (34.9)	61.4 (39.3)	61.7 (43.7)	59.5 (39.4)	
Median (Q1, Q3)	52.4 (31.5, 70.4)	56.2 (33.5, 79.8)	57.0 (29.0, 81.3)	54.6 (31.7, 77.1)	
Missing	100 (35.7%)	103 (37.5%)	99 (36.4%)	302 (36.5%)	
**Glucose**					**<0.001**
Mean (SD)	103 (30.0)	113 (36.8)	132 (61.0)	116 (46.1)	
Median (Q1, Q3)	97.0 (88.3, 110)	103 (92.0, 121)	111 (96.0, 148)	102 (91.0, 124)	
Missing	26 (9.3%)	18 (6.5%)	20 (7.4%)	64 (7.7%)	
**Glucose above 200 mg/dL**	5 (1.8%)	6 (2.2%)	25 (9.2%)	36 (4.4%)	**<0.001**
**Comorbidities**					
Diabetes mellitus type 2	21 (7.5%)	46 (16.7%)	83 (30.5%)	150 (18.1%)	**<0.001**
Coronary artery disease	25 (8.9%)	55 (20.0%)	76 (27.9%)	156 (18.9%)	**<0.001**
Cerebrovascular disease	6 (2.1%)	8 (2.9%)	25 (9.2%)	39 (4.7%)	**<0.001**
Peripheral artery disease	6 (2.1%)	8 (2.9%)	22 (8.1%)	36 (4.4%)	**<0.001**
Chronic kidney disease	12 (4.3%)	18 (6.5%)	38 (14.0%)	68 (8.2%)	**<0.001**
Dyslipidemia	52 (18.6%)	80 (29.1%)	109 (40.1%)	241 (29.1%)	**<0.001**
Hypertension	83 (29.6%)	138 (50.2%)	187 (68.8%)	408 (49.3%)	**<0.001**

**Table 2:** Overview of carotid-femoral pulse wave velocity (cfPWV) and thyroid hormone level stratified age groups. SD = standard deviation; Q = quartile.

Age Group (Years)	18–53 (*n* = 315)	53–70 (*n* = 310)	70–100 (*n* = 293)	Overall (*n* = 918)
**cfPWV**				
Mean (SD)	8.26 (1.97)	10.1 (2.49)	12.1 (3.57)	9.99 (3.10)
Median (Q1, Q3)	8.00 (7.10, 9.18)	9.70 (8.50, 11.3)	11.9 (10.4, 14.1)	9.40 (7.90, 11.8)
Missing	9 (2.9%)	26 (8.4%)	56 (19.1%)	91 (9.9%)
**TSH**				
Mean (SD)	1.48 (1.10)	2.00 (3.41)	2.04 (3.43)	1.83 (2.85)
Median (Q1, Q3)	1.25 (0.818, 1.85)	1.40 (0.860, 2.07)	1.29 (0.840, 2.14)	1.28 (0.835, 2.00)
Missing	87 (27.6%)	97 (31.3%)	71 (24.2%)	255 (27.8%)
**fT3**				
Mean (SD)	3.01 (1.18)	4.64 (26.2)	2.47 (0.783)	3.34 (14.7)
Median (Q1, Q3)	2.90 (2.57, 3.31)	2.77 (2.42, 3.09)	2.45 (2.05, 2.81)	2.73 (2.32, 3.10)
Missing	101 (32.1%)	118 (38.1%)	87 (29.7%)	306 (33.3%)
**fT4**				
Mean (SD)	1.20 (0.220)	2.11 (12.1)	1.43 (0.705)	1.57 (6.86)
Median (Q1, Q3)	1.20 (1.06, 1.33)	1.24 (1.12, 1.43)	1.33 (1.19, 1.54)	1.25 (1.11, 1.43)
Missing	88 (27.9%)	98 (31.6%)	73 (24.9%)	259 (28.2%)

## Data Availability

The data presented in this study are available on request from the corresponding author in accordance with national law and organizational regulations.

## Supplementary Materials

The following supporting information can be downloaded at: https://www.mdpi.com/article/10.3390/medicina61050812/s1, Figure S1: Association of carotid-femoral pulse wave velocity (cfPWV) with typical cardiovascular comorbidities; Figure S2: Association of carotid-femoral pulse wave velocity (cfPWV) with iPTH tertiles in subgroups based on comorbidities; Figure S3: Association of carotid-femoral pulse wave velocity (cfPWV) with glucose tertiles in subgroups based on comorbidities; Figure S4: Association of carotid-femoral pulse wave velocity (cfPWV) with glucose levels above 200 mg/dl in subgroups based on comorbidities.
