# Supplementary material for: Associations of Thyroid and Parathyroid Hormones with Arterial Stiffness in Emergency Department Patients: A Prospective Cross-Sectional Study
Source: Medicina (Kaunas). 2025 Apr 28;61(5):812. doi: 10.3390/medicina61050812 (PMC12113468; doi:10.3390/medicina61050812)
Supplement: Supplementary file 1 [file medicina-61-00812-s001.zip › medicina-3548917-supplementary.pdf]

medicina

MDPI

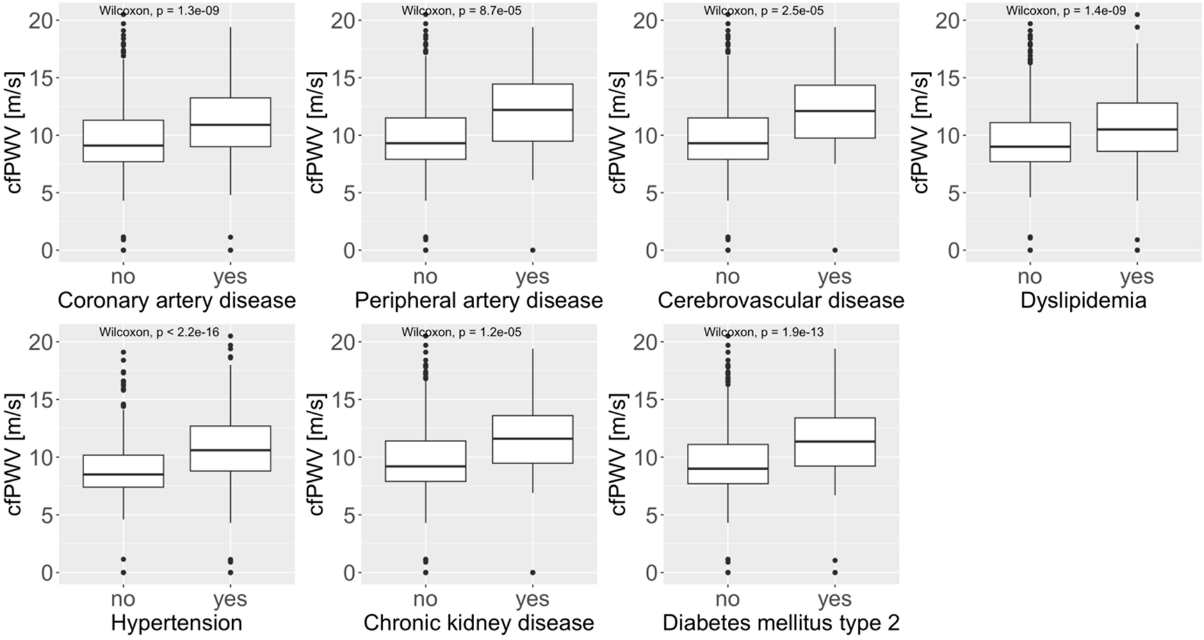

Supplemental Figure S1: *Association of carotid-femoral pulse wave velocity (cfPWV) with typical cardiovascular comorbidities.*

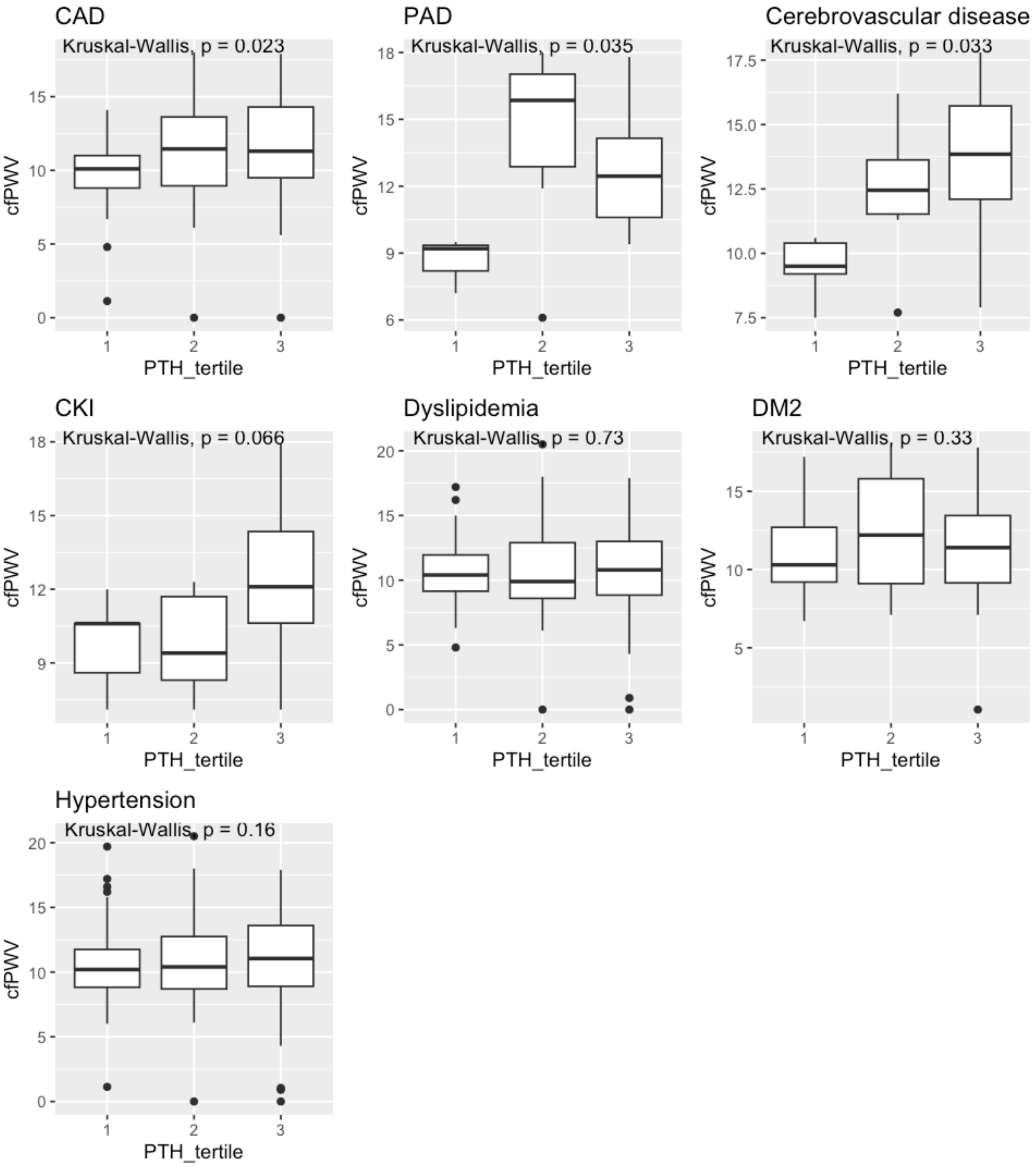

Supplemental Figure S2: *Association of carotid-femoral pulse wave velocity (cfPWV) with iPTH tertiles in subgroups based on comorbidities.*

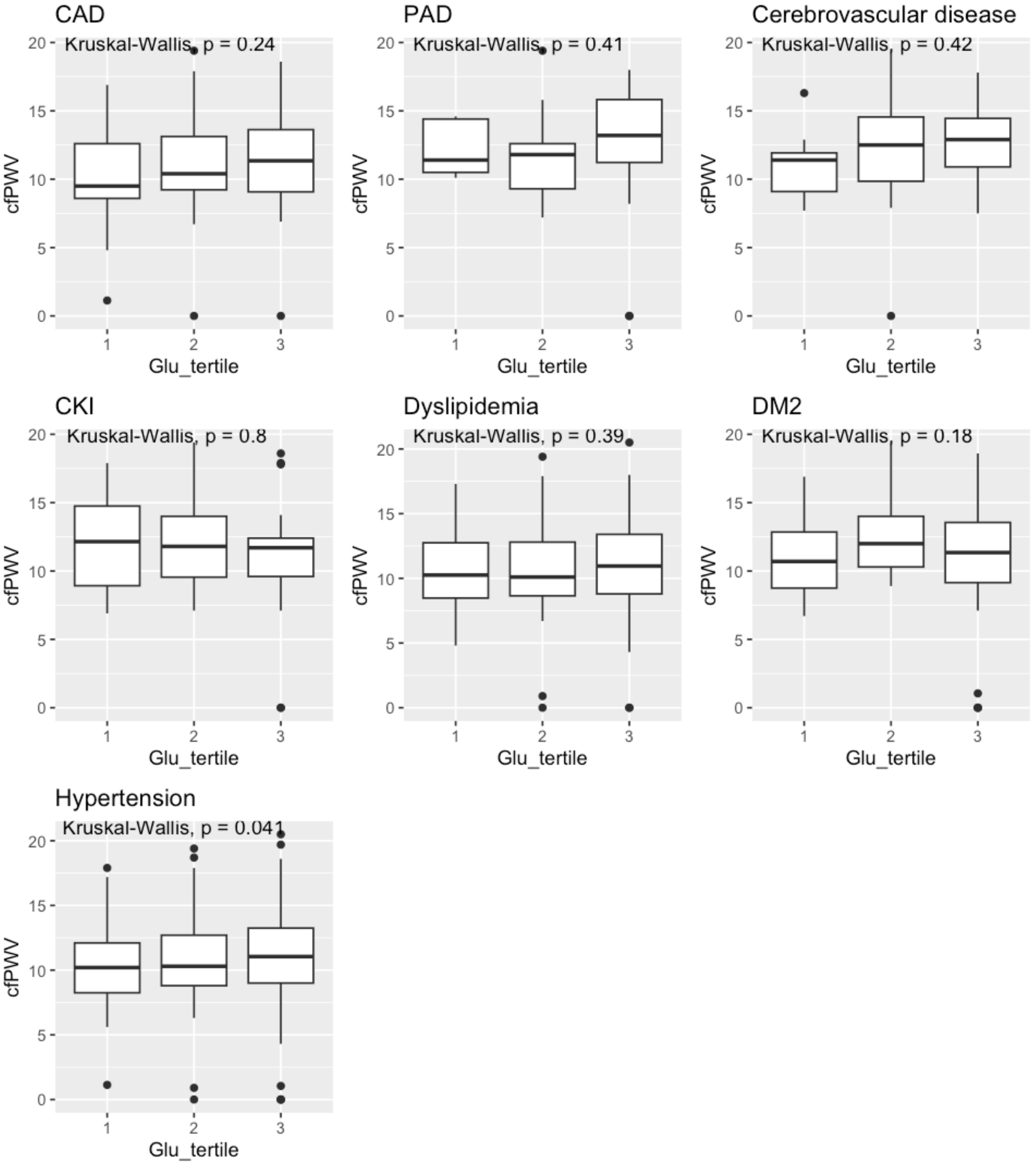

***Supplemental Figure S3:** Association of carotid-femoral pulse wave velocity (cfPWV) with glucose tertiles in subgroups based on comorbidities.*

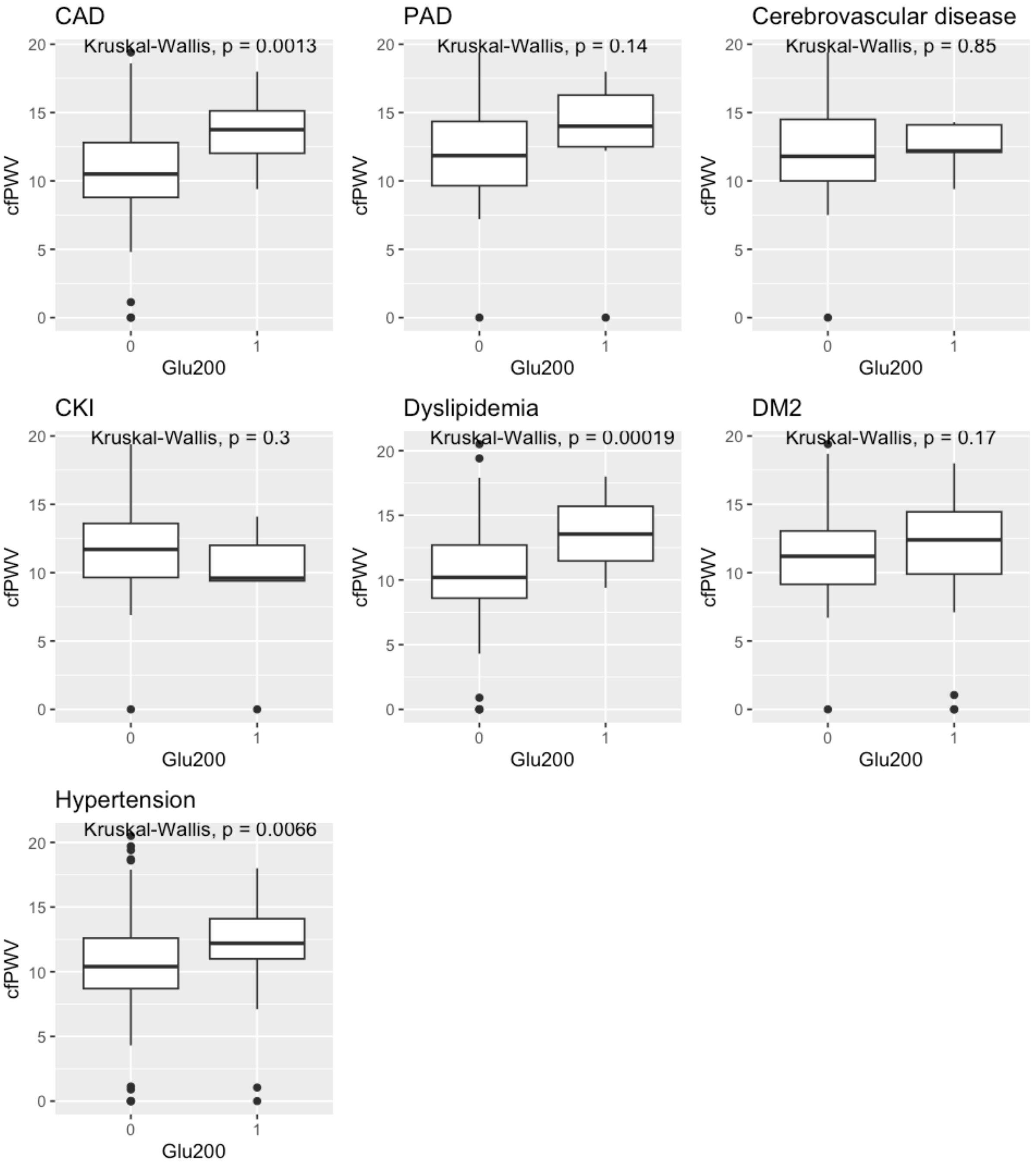

Supplemental Figure S4: *Association of carotid-femoral pulse wave velocity (cfPWV) with glucose levels above 200mg/dl in subgroups based on comorbidities.*

Disclaimer/Publisher's Note: The statements, opinions and data contained in all publications are solely those of the individual author(s) and contributor(s) and not of MDPI and/or the editor(s). MDPI and/or the editor(s) disclaim responsibility for any injury to people or property resulting from any ideas, methods, instructions or products referred to in the content.
